# Wearable Adaptive Resistance Training Improves Ankle Strength, Walking Efficiency and Mobility in Cerebral Palsy: A Pilot Clinical Trial

**DOI:** 10.1109/OJEMB.2020.3035316

**Published:** 2020-11-02

**Authors:** Benjamin C. Conner, Nushka M. Remec, Elizabeth K. Orum, Emily M. Frank, Zachary F. Lerner

**Affiliations:** B. C. Conner is with the University of Arizona College of Medicine - Phoenix, Phoenix, AZ, USA. N. M. Remec is with Phoenix Children’s Hospital, Phoenix, AZ, USA. E. K. Orum and E. M. Frank are with the College of Health Solutions, Arizona State University, Tempe, AZ, USA. Z. F. Lerner is with the Mechanical Engineering Department, Northern Arizona University, Flagstaff, AZ, USA and also the Department of Orthopedics, University of Arizona College of Medicine – Phoenix, Phoenix, AZ, USA

**Keywords:** cerebral palsy, exoskeleton, plantar flexor, neurorehabilitation

## Abstract

**Goal:**

To determine the efficacy of wearable adaptive resistance training for rapidly improving walking ability in children with cerebral palsy (CP).

**Methods:**

Six children with spastic CP (five males, one female; mean age 14y 11mo; three hemiplegic, three diplegic; Gross Motor Function Classification System [GMFCS] levels I and II) underwent ten, 20-minute training sessions over four weeks with a wearable adaptive resistance device. Strength, speed, walking efficiency, timed up and go (TUG), and six-minute walk test (6MWT) were used to measure training outcomes.

**Results:**

Participants showed increased average plantar flexor strength (17 ± 8%, p = 0.02), increased preferred walking speed on the treadmill (39 ± 25%, p = 0.04), improved metabolic cost of transport (33 ± 9%, p = 0.03), and enhanced performance on the timed up and go (11 ± 9%, p = 0.04) and six-minute walk test (13 ± 9%, p = 0.04).

**Conclusions:**

The observed increase in preferred walking speed, reduction in metabolic cost of transport, and improved performance on clinical tests of mobility highlights the potentially transformative nature of this novel therapy; the rate at which this intervention elicited improved function was 3 – 6 times greater than what has been reported previously.

## Graphical Abstract

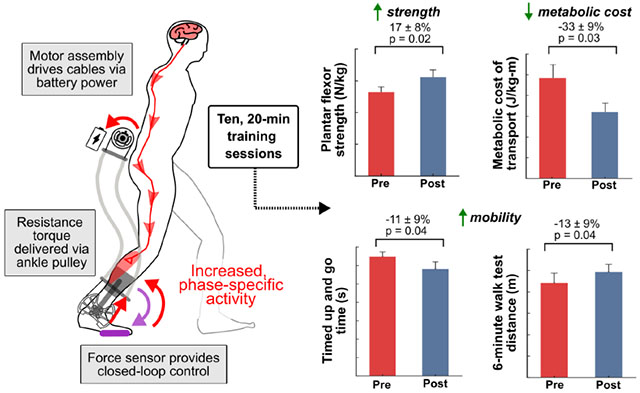

**Resist to restore:** A lightweight, untethered ankle exoskeleton device was used to restore strength and improve walking function in six children with cerebral palsy. Ten, 20-minute training session with wearable adaptive resistance, which provided proportional resistance to plantar flexion during the stance phase of gait, led to significant improvements in ankle plantar flexor strength, metabolic cost of transport, and performance on tests of agility and endurance.

## I. Introduction

AFFECTING 17 million people globally [1], cerebral palsy (CP) is a movement disorder caused by injury to the developing brain, characterized by deficits in strength [2] and neuromuscular coordination [1]. These deficits lead to inefficient movement patterns that elevate the energy cost of walking by 2 – 3 times that of unimpaired individuals [3] and reduce walking speed by 10 – 40% [4]. Physical activity, an essential stimulus for healthy development, is dramatically lower for children with CP [5] and is negatively associated with the higher energy cost of movement [6]. While surgical and pharmacological interventions successfully contribute to the management of the most severe presentations of CP [7], lifelong walking disability remains for nearly all individuals affected by CP. Addressing the fundamental neuromuscular deficits preventing efficient movement, therefore, is an essential task in the care of individuals with CP.

Treatment strategies for improving the gait of individuals with CP typically consist of a combination of orthopedic surgery, physical therapy, and assistive devices [8]. Orthopedic surgery, while effective in improving lever arm dysfunction by the modification of muscle and tendon origin and insertion points, is not able to directly address the muscle weakness [2] and neuromuscular coordination deficits [1] seen in CP. In addition, physical therapy has limited evidence for improving mobility, with interventions such as strength training lacking the task specificity necessary for improving walking function [9]. Recently, treadmill training with partial body-weight support has gained traction as a potential task-specific intervention for improving mobility in CP [10]. However, a potential weakness of this training modality is the relative unloading, which could lead to muscle inactivation [11] and atrophy [12] over time. Additionally, the equipment required for this type of training is expensive and tethered [13], leading to limited access for patients.

There is a vital need for an effective and widely-available intervention capable of increasing the dose of task-specific gait therapy for children with CP. To meet this need, a wearable robotic device was recently developed to address deficits in muscle recruitment and reinforce neuromuscular control patterns that may produce lasting improvements in locomotor function [14]. Utilizing onboard sensors and a closed-loop control strategy that responds immediately to user input, this novel training modality provides perfectly synchronized resistance to re-train ankle plantar flexor muscle function during the stance phase of walking. This device, unlike passive gait training interventions, provides an opportunity to monitor user engagement in real-time, allowing for the immediate performance feedback necessary for increasing task skill [15].

The goal of this foundational clinical trial was to determine the efficacy of the first intervention using wearable adaptive resistance for improving gait in children with spastic CP. We hypothesized that this neuromuscular gait re-training intervention would strengthen the plantar flexors, increase preferred gait speed, and reduce the energy cost of walking. We also hypothesized that these improvements would translate to better performance on clinically validated tests of walking function.

## II. Methods

### A. Participants

Participants diagnosed with spastic hemi- or diplegic CP were recruited from physical therapy clinics in the local area. Inclusion criteria included Gross Motor Function Classification System (GMFCS) levels I – II, ability to walk with or without a walker for at least six minutes, age between 10 – 21 years, and the ability to follow simple directions. Exclusion criteria included orthopedic surgery within the past six months or any conditions that would prevent safe participation. This study was approved by Northern Arizona University’s Institutional Review Board (#986744) and registered at ClinicalTrials.gov (NCT04119063). Informed written consent was provided by a parent or legal guardian for each participant after the nature and possible consequences of the study was explained; participants provided verbal assent. Additionally, informed consent to publish identifying information was obtained from all the participants and their parents or legal guardian.

### B. Wearable adaptive resistance device

**Fig. 1.**
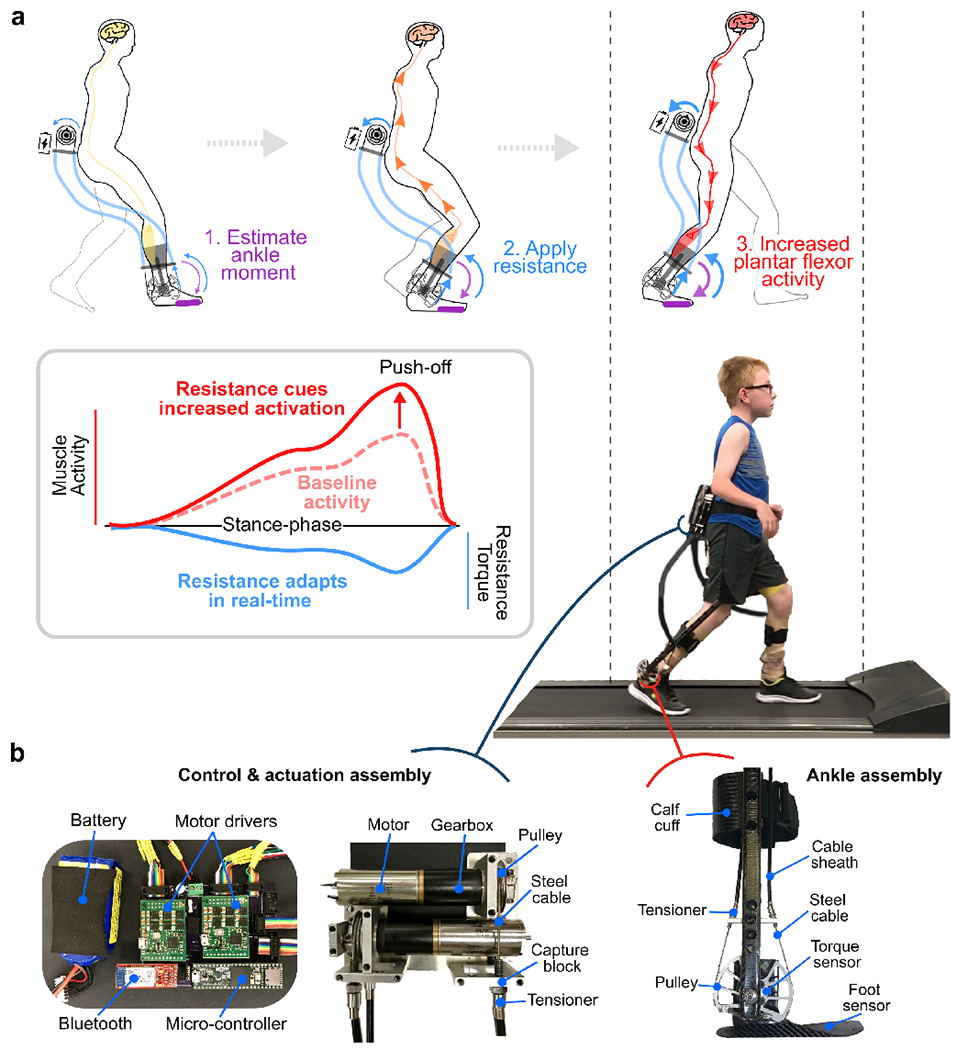
A wearable adaptive resistance device for neuromuscular gait re-training: a) Using the real-time estimation of biological ankle moment via embedded foot sensors, a proportional level of resistance to plantar flexion is applied that serves as an afferent signal for increasing motor unit recruitment of the plantar flexors, resulting in increased push-off for forward progression; b) Components of the wearable adaptive resistance device worn by all participants while training. The wireless robotic device included a waist-mounted control and actuation assembly, and carbon fiber ankle assemblies that were customized for each participant.

Each participant was outfitted with a battery-powered, custom fit, lower limb ankle exoskeleton device (Fig. 1). The robotic device consisted of an actuation & control assembly worn at the waist, and ankle assemblies worn bilaterally on the legs (Fig. 1B). Motors remotely actuated the carbon fiber ankle assemblies via Bowden cables to generate ankle torque in the sagittal plane. Using custom force sensors placed under the forefoot, the ankle moment was estimated and a proportional level of resistance to plantar flexion was applied bilaterally in real-time during each stance phase. Exoskeleton resistance torque was measured bilaterally at the ankle assembly level using a torque sensor that was aligned with the ankle joint at the lateral malleolus. Users started with a calibration procedure that calculated the average peak ankle moment while walking. A resistance level could then be set (i.e., 0.1 Nm/kg), whereby this magnitude of resistance would be applied proportional to the instantaneous, estimated ankle moment. In other words, when peak ankle moment was reached (typically occurring in late stance), 0.1 Nm/kg would be applied. If only 50% of peak ankle moment was estimated (i.e., early stance), only 0.05 Nm/kg would be applied. If 0% of peak ankle moment was estimated (i.e., swing phase), 0 Nm/kg would be applied. This proportional application of resistance to plantar flexion occurred instantaneously and throughout the entire gait cycle for both ankles.

The typical user response to overcome the resistance was to increase activation of the ankle plantar flexors and decrease activation of the ankle dorsiflexors (reduce co-contraction) (Fig. 1) [14]. The closed-loop control scheme was designed to maximize neuromuscular engagement by being immediately responsive (i.e., adaptive) to user input. The device was controlled via a custom MATLAB graphical user interface (v2019b, Natick, MA, USA), and weighed 1.75 kg. Additional details on the exoskeleton design and adaptive ankle resistance control scheme can be found in [14].

### C. Assessments

Participants completed twelve total visits: a pre-assessment (first visit), ten training sessions, and a post-assessment (last visit). On the first visit, participants were evaluated by a licensed physical therapist to determine baseline physical characteristics, GMFCS level, and walking pattern. The following outcome measures were then evaluated on the first and last visit: plantar flexor strength, preferred treadmill walking speed, metabolic cost of transport, timed up and go (TUG) time, and six-minute walk test (6MWT) distance.

Plantar flexor strength was evaluated using a maximum voluntary contraction (MVC). Lying supine, participants were instructed to push as hard as possible for 3 seconds into a handheld dynamometer, and an average of three trials was recorded. Values were then averaged between limbs and normalized to body mass. Using the physical therapist’s assessment, MVC measures, and input from parents/guardian, a more-affected and less-affected side were determined for training purposes.

To assess the metabolic cost of transport, a metabolic mask was worn for collecting oxygen and carbon dioxide levels (TrueOne 2400, Parvo Medics, Salt Lake City, UT, USA). First, these expired gases were recorded during quiet sitting and standing. Next, participants walked on a treadmill at a self-selected speed while being spotted by a laboratory technician. Preferred walking speed was identified by asking participants to choose a speed that they would normally walk at while at school or home. The treadmill speed was then increased and decreased to confirm preferred walking speed. Participants walked for approximately six minutes, or until oxygen consumption plateaued, indicating a stabilized metabolic cost of walking.

Two reliable, clinical measures for children with CP, the TUG [16] and 6MWT [17], were used to measure the effect of training on mobility. For both measures, we followed standard testing procedures [16], [17]. S4 presented behavioral issues and was not able to complete the 6MWT without periods of running on his final assessment, which was out of compliance with instructions to only walk.

### D. Training

Participants completed ten training sessions over four weeks. The first session began with resistance set at 0.025 – 0.075 Nm/kg while walking at a preferred treadmill walking speed (Fig. 1). When necessary, instruction was given to participants while they walked to “push against the resistance”, where the focus was on their more-affected side. Using real-time feedback of the estimated biological ankle moment and exoskeleton ankle torque, the research team was able to assess how well participants were reaching the prescribed level of resistance. Training included 20 minutes of walking per visit, separated by one to two rest breaks depending on participant preference. At the end of each session, participants were asked to rank their level of soreness on the following scale: None, Mild, Moderate, Severe, and Very Severe. Resistance was increased by 0.5 – 1 Nm per session if participants both 1) reached their prescribed level of resistance > 50% of the training session, and 2) had a perceived level of soreness between None and Moderate. All six subjects completed ten training sessions.

### E. Data processing & statistical analysis

For analysis of metabolic data, we identified areas of “steady state” for both standing and walking using Kendall’s tau-b approach [18], which is able to categorize time series data as rising, falling, or stable. The null hypothesis of this analysis was that a data point falls within a steady state window, and rejection of the null hypothesis indicated non-steady data. This technique for determining which data points can be considered steady state for a condition was found to contribute to a fivefold reduction in variability of measured oxygen costs while walking for individuals with CP [18]. Brockway’s standard equation was used to determine metabolic cost for each steady-state region [19]. Net metabolic cost (W) was calculated by subtracting the metabolic cost of quiet standing from the metabolic cost of walking. To normalize to body size and differences in walking speed, metabolic cost was then divided by body mass (kg) and walking speed (m/s) for a final measure of body-mass-normalized metabolic energy required to walk a unit distance (i.e., metabolic cost of transport, J/kg-m).

We assessed all outcome measure data for normality and the presence of outliers. Normality was tested using the Kolmogorov-Smirnov test with small sample Lilliefors correction [20]. Outliers were defined as any data point 1.5 times the interquartile range below the first quartile or above the third quartile. Data falling within this outlier definition were removed for statistical comparison and calculation of means, and not included in figures unless explicitly noted. We assessed our *a priori* hypotheses using two-tailed paired t-tests, and accounted for multiple comparisons using a Holm-Bonferroni correction. Significance level was set at α < 0.05. Cohen’s d (d) was used to calculate effect size, where 0.2 was considered a small effect, 0.5 a medium effect, and 0.8 a large effect [21].

## III. Results

Six participants (five males, one female) with mild-to-moderate gait impairment from CP (all independent ambulators) completed pre- and post-assessments and ten training visits (Table I). All data assessed in the statistical comparisons were normally distributed. S3’s pre-assessment metabolic cost of transport, an unrealistic 16.9 J/kg-m, met the definition of an outlier and was subsequently removed from any statistical analyses.

**Fig. 2.**
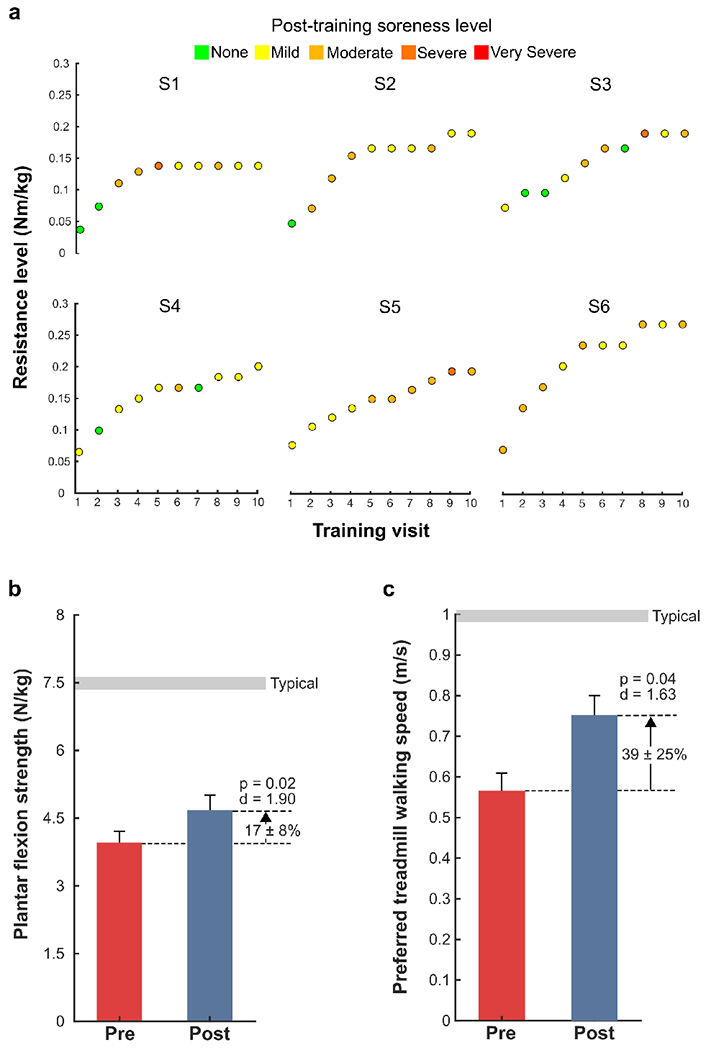
Training progression, strength and speed outcomes: a) Training progression with indication of the resistance level (Nm/kg) for each of the ten training visits (v2 – v11) and the resulting soreness level from each session: None (green), Mild (yellow), Moderate (light orange), Severe (dark orange), and Very Severe (red); b) Maximum voluntary contraction values for the plantar flexors, presented as the average between both limbs and normalized to body mass; c) Preferred walking speed (m/s) on the treadmill.

There was a significant increase in average plantar flexion strength by 17 ± 8% after training (p = 0.02, d = 1.90; Fig. 2b, Table II). Preferred walking speed on the treadmill increased by 39 ± 25% after training (p = 0.04, d = 1.63; Fig. 2c, Table II). Level of resistance reached by visit ten ranged from 0.14 – 0.27 Nm/kg. The average soreness level for all participants and visits was Moderate; the range of soreness levels spanned None – Severe, with no participants reaching a Very Severe level (Fig. 2a).

**Fig. 3.**
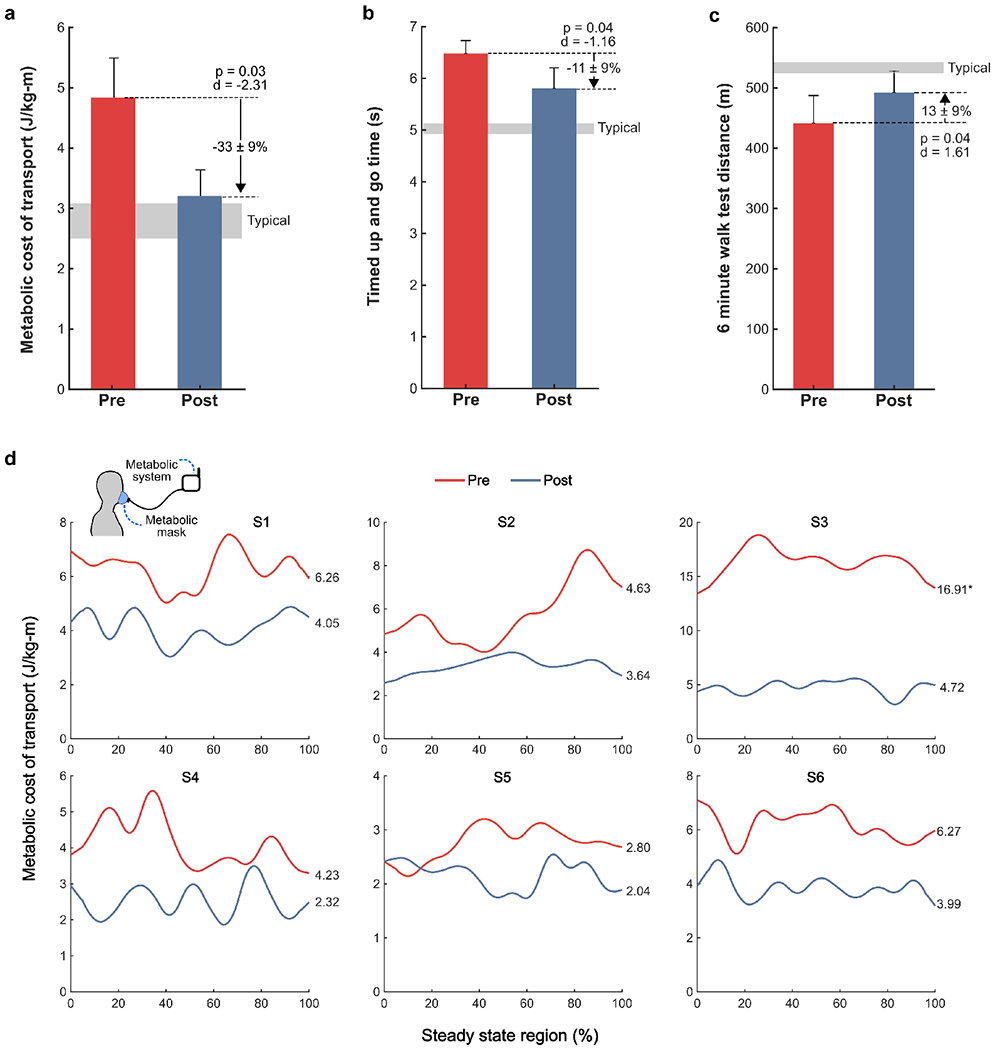
Walking efficiency and clinical mobility outcomes. Pre- (red) and post-assessment (blue) a) group-level metabolic cost of transport, representing the metabolic cost per unit distance of walking while participants walked on a treadmill at their within-visit preferred speed, b) timed up and go (TUG) times, averaged over 3 trials, c) six-minute walk test (6MWT) distance, and d) individual, steady-state metabolic data. *Indicates outlier, excluded from statistical analysis.

Metabolic cost of transport, evaluated at a participant’s within-visit preferred walking speed on the treadmill, decreased from pre- to post-assessments by 33 ± 9% (p = 0.03, d = −2.31; Fig. 3a,3d).

TUG times decreased by 11 ± 9% (p = 0.04, d = −1.16; Fig. 3b, Table II) and 6MWT distances increased by 13 ± 9% (p = 0.04, d = 1.61; Fig. 3c, Table II).

## IV. Discussion

Wearable adaptive ankle resistance was designed to provide precise, perfectly synchronized resistance within the functional task of walking. The novel closed-loop control scheme that responded in real-time to user input acted as a neuromuscular cue to elicit improved function of the ankle plantar-flexors. Adaptive ankle resistance training of ten, 20-minute sessions over four weeks was safe and effective for children and adolescents with CP (Supp Video 1, Supp Video 2).

We found that participants’ soreness levels ranged from None – Severe. Importantly, “Severe” soreness was only noted three times, representing <5% of questionnaire responses across the study, and no participants indicated a “Very Severe” level. Soreness level did not appear to negatively affect training efficacy with all participants safely completing the entire 20-minute duration of all 10 of their training sessions. In fact, the protocol was designed to elicit moderate soreness after each session, increasing resistance, if necessary. The training progression for participants depended on both soreness level and the ability to match the targeted level of resistance for more than 50% of the training session. Active engagement is a critical aspect of neuromuscular re-training [22]. Therefore, our ability to assess engagement with resistance was an essential aspect of this training modality. Using real-time displays of exoskeleton resistance and estimated biological ankle moment, we were able to track “performance” by analyzing how well a participant’s biological ankle moment matched the prescribed level of resistance. This helped to prevent passivity while training, as we could adjust resistance or further instruct participants based on their ability to engage with resistance. We observed that participants were more likely to be sore with a higher percentage of gait cycles reaching the prescribed resistance level, supporting our criteria for increasing the level of resistance.

Our finding of increased average plantar flexion strength may be a positive indicator of the tissue-level changes that are possible with this wearable resistance training modality. Plantar flexion strength is significantly associated with walking ability for individuals with CP [23], who are weaker than their typically developing peers [2]. Strength is a necessary building block for function, and the increases in strength with training likely contributed to the improved walking efficiency and performance we observed.

Metabolic cost of walking, an important, inversely-related indicator of physical activity levels in children with CP [6], significantly improved after the intervention. We observed a significant reduction with a very large effect size in metabolic cost of transport (−33%, p = 0.03, d = −2.31) when compared to the metabolic cost of transport during the pre-assessment (Fig. 3a,3d). This reduced metabolic cost of transport occurred at participants’ final preferred speed, which was 39% faster than their pre-assessment speed (p = 0.04, d = 1.63). These results suggest that following the intervention, participants could walk 39% faster while using 33% less energy per unit distance, on average.

To confirm that changes in metabolic cost of transport could translate to improved performance on tests of speed and endurance, we included the TUG and 6MWT as part of the pre- and post-assessment; outcomes from both improved. Time on the TUG test, representing performance in agility and speed, decreased 11%. The TUG requires participants to walk as quickly as possible, and involves a sit to stand and stand to sit movement that requires careful control and good functional strength beyond the task of walking [24]. The observed 13% improvement in 6MWT, a test of endurance, may be attributed to the improved walking efficiency following ankle resistance training. This is a particularly promising finding given the significant association between 6MWT performance and physical activity levels in children with CP [25].

Compared to treadmill training alone, our wearable resistance intervention elicited similar or greater improvements in a fraction of the training time. Provost and colleagues studied the effect of intensive body weight-supported treadmill training for six individuals with CP, GMFCS level I, ages 6-14 [26]. The intervention consisted of 30 minutes, twice daily training with 0-30% weight support over 12 total sessions. Effects of body weight-supported treadmill training on walking efficiency, assessed using the Energy Expenditure Index (EEI), an indirect measure of energy expenditure based on heart rate, improved for all six children (d = −1.24, calculated from the individual participant data provided), yet 6MWT distance did not improve. Despite the shorter training period in our study (200 minutes versus 720 minutes), we still observed clinically-meaningful reductions in metabolic cost of transport (−33%) with an effect size of −2.31, and did observe improvements in 6MWT distance (13%). In a study by Aviram and colleagues [27], 43 individuals with spastic CP, GMFCS levels II – III, ages 14 – 21 years underwent 30 bi-weekly, 40-minute treadmill training sessions under the supervision of a physical therapist with progressive increases in treadmill speed. After training, individuals had a 10.1% decrease in TUG time (−1.21 ± 0.40 s) and a 12.1% increase in 6MWT distance (29.1 ± 6.9 m). Individual subject data were not provided preventing comparison of effect size, nor was energy expenditure assessed. Notably, the total accumulated training time was approximately 1200 minutes, six times the duration of our intervention.

### A. Study limitations

There are limitations of this study worth noting. First, this study lacked a control group. Therefore, we rigorously compared our findings to relevant intervention studies comprised of only treadmill walking. We found that our adaptive resistance intervention resulted in similar or greater improvements following a dramatically shorter intervention duration. The next step, after these promising findings, is a randomized controlled trial (RCT) that will include a dose-matched control group.

Second, this study had a small sample size. Despite this limitation, we observed mainly large and very large effect sizes for our outcome measures. Participants in this study were also limited to an age of at least 10 years old and GMFCS levels I – II. It is important to consider these participant characteristics because it is possible that training effects would look different for younger individuals with CP, when neuroplasticity may be higher [28], and for individuals with lower functional levels, such as GMFCS level III, where there may be more room for improvement in strength [29] and gait efficiency [30]. As a pilot study, this inclusion criteria allowed us to be confident in the feasibility of this training intervention, and our findings now provide justification for broadening the inclusion criteria for future explorations of this training modality.

Third, verbal instruction was provided during each functional ankle resistance training session. Therefore, the impact of verbal instruction on our outcomes is unknown. Real-time biofeedback has shown promise in gait training for children with CP [31], and will be studied as an automated approach for providing feedback during our future implementations of adaptive resistance training. Finally, to better understand the mechanisms of improved walking efficiency with wearable adaptive resistance training, future work should evaluate changes in neuromuscular control of the lower limbs.

### B. Conclusion

Wearable adaptive resistance training was found to be a feasible and effective modality for improving locomotor performance in individuals with CP who had life-long, deeply engrained walking patterns. A short, four-week intervention was able to significantly improve ankle strength, walking efficiency and performance on clinical tests of speed and endurance. We expect that this intervention may be suitable for other patient populations affected by neuromuscular impairment because it aligns with the guiding principles of neuroplasticity – task-specificity and top-down active engagement [15]. This relatively low-cost, battery-powered, and wearable intervention was designed for translation to both clinical practice and personal use at home, and as an untethered device, training can take place under a variety of contexts, including overground walking [32]. The inherent accessibility of this wearable intervention provides individuals with the opportunity to significantly increase the frequency of targeted neuromuscular rehabilitation.

## Supplementary Material

Supp Video 1. Subject 2 training progression with wearable adaptive resistance. Supp Video 1. Subject 2 training progression with wearable adaptive resistance.

Supp Video 2. Subject 6 training progression with wearable adaptive resistance. Supp Video 2. Subject 6 training progression with wearable adaptive resistance.

## Figures and Tables

**TABLE I:** PARTICIPANT CHARACTERISTICS

	Gender	Age (y:m)	GMFCS^a^	Distribution	Gait type^b^
S1	M	16:11	II	Diplegic	Crouch
S2	M	13:11	II	Hemiplegic	Crouch
S3	M	15:11	II	Diplegic	Winters type II
S4	M	14:10	I	Hemiplegic	Crouch
S5	F	16:5	I	Hemiplegic	Stiff knee
S6	M	11:8	II	Diplegic	Crouch

a GMFCS: Gross Motor Function Classification System

b Gait type: ‘Crouch’ gait defined by those parameters set by Gage et al [33] and ‘Winters type’ defined by those parameters set by Winters et al [34].

**TABLE II:** Strength and mobility outcomes

	Plantar flexor strength (N/kg)^a^*		Preferred treadmill walking speed (m/s)*		Timed up and go (s)*		Six-minute walk test (m)*	
	Pre	Post	Pre	Post	Pre	Post	Pre	Post
S1	3.79	4.73	0.58	0.89	6.60	5.86	589	597
S2	2.57	2.74	0.45	0.63	7.04	5.71	374	437
S3	4.73	5.59	0.40	0.63	7.30	7.65	349	400
S4	4.16	4.46	0.67	0.67	6.28	5.47	-	-
S5	3.65	4.52	0.72	0.85	5.75	5.29	505	545
S6	4.71	5.75	0.49	0.80	5.91	4.87	391	485

Median	3.97	4.62	0.53	0.73	6.44	5.59	391	485

a Indicates the maximum voluntary contraction value for the plantar flexors, presented as the average between limbs and normalized to body mass.

Note: S4 was not able to follow the instructions to “walk only” and thus, could not complete the six-minute walk test.

* p < 0.05

## Impact Statement

Wearable adaptive resistance has the potential to be a transformative intervention for improving mobility in children with CP, with a low-cost and mobile design for increased accessibility and therapy frequency.
